# Translation and cross-cultural adaptation of the Composite Abuse Scale into Brazilian Portuguese

**DOI:** 10.11606/s1518-8787.2022056004196

**Published:** 2022-11-18

**Authors:** Raiza Wallace Guimaraes da Rocha, Daniel Canavese de Oliveira, Vitor Adriano Liebel, Patricia Helena Rubens Pallu, Kelsey Lee Hegarty, Marcos Claudio Signorelli

**Affiliations:** I Universidade Federal do Paraná Curitiba PR Brasil Universidade Federal do Paraná. Programa de Pós-Graduação em Saúde Coletiva. Curitiba, PR, Brasil; II Universidade Federal do Rio Grande do Sul Departamento de Saúde Coletiva Porto Alegre RS Brasil Universidade Federal do Rio Grande do Sul. Departamento de Saúde Coletiva. Porto Alegre, RS, Brasil; III V.A. Liebel Linguistic Solutions Curitiba PR Brasil V.A. Liebel Linguistic Solutions. Curitiba, PR, Brasil; IV Colégio Positivo Junior Curitiba PR Brasil Colégio Positivo Junior. Curitiba, PR, Brasil; V University of Melbourne The Royal Women's Hospital Department of General Practice Melbourne VIC Australia University of Melbourne. Department of General Practice. The Royal Women's Hospital. Melbourne, VIC, Australia; VI Universidade Federal do Paraná Departamento de Saúde Coletiva Setor de Ciências da Saúde Curitiba PR Brasil Universidade Federal do Paraná. Setor de Ciências da Saúde. Departamento de Saúde Coletiva. Curitiba, PR, Brasil

**Keywords:** Violence Against Women, Intimate Partner Violence, Surveys and Questionnaires, Translating, Cross-Cultural Comparison

## Abstract

**OBJECTIVE:**

To perform the translation and cross-cultural adaptation from English into Brazilian Portuguese of the Composite Abuse Scale, an instrument that identifies and quantifies intimate partner violence.

**METHODS:**

This study is based on the strict implementation of its previously published protocol, which consists of ten steps: (a) conceptual analysis; (b) double-blind translation; (c) comparison and first reconciled version of the two translations; (d) back-translation; (e) review of the back-translation by the developer and second reconciled version; (f) expert committee review (n = 6); (g) comparison of expert reviews and third reconciled version; (h) cognitive interviews with women from the *Casa da Mulher Brasileira* in Curitiba (n = 15); (i) assessments of user perceptions and final reconciliation; and (j) submission of the final version of the questionnaire to the developer.

**RESULTS:**

The implementation of the 10 steps of the protocol allowed the idiomatic, semantic, conceptual and experiential equivalences of the Composite Abuse Scale, incorporating suggestions and criticisms from the different participants of the process. Participants included the developer, professional translators, researchers specialized on the subject, women in situation of intimate partner violence, and professionals who provide care to them. Experts and cognitive interviews with women were instrumental in ensuring equivalence, and facilitating the understanding, including: (1) adaptation of the term “intimate relation” to “affective or conjugal relation”; (2) substitution of enclisis for proclisis cases in 20 items; (3) adoption of gender-neutral language, allowing its use in heterosexual, bisexual, and same-sex relations; (4) materialization of an instrument of scientific rigor and self-applicable, which may help women to visualize the situations of abuse in their relations.

**CONCLUSIONS:**

The translation and cross-cultural adaptation process of the Composite Abuse Scale resulted in the Composite Abuse Scale Brazilian Portuguese Version, a 30-item self-applicable instrument, capable to identify and quantify intimate partner violence, its frequency, severity and typologies (physical, emotional, harassment and severe combined violence).

## INTRODUCTION

Intimate partner violence (IPV) is a global public health problem^1,2^. Estimates from the World Health Organization (WHO) indicate that 23% of Brazilian women have experienced IPV^3^. Between 2011 and 2017, most of gender-based violence notifications in the Unified Health System (SUS) were for intimate partner violence^4–6^.

Eliminating IPV is one of the Sustainable Development Goals for 2030^7^, of which Brazil is a signatory. The country has made efforts^8,9^ to prevent this type of violence, but challenges remain^10^. In 2021, specific legislation was approved to typify psychological violence against women^11^, estimated as the most prevalent subtype of intimate partner violence^12^. This, however, is the most difficult violence to identify when compared to the other subtypes of IPV (physical or sexual), and is described as the most striking to women's health^12,13^. Therefore, it is necessary to have reliable instruments^14,15^ to identify and measure this type of issue (psychological violence), not disregarding the other subtypes of IPV.

Identifying and quantifying IPV and its subtypes, using comparable and scientifically rigorous methods contributes to monitoring and supporting decision-making^16^. The Composite Abuse Scale (CAS) is a scale developed in Australia, internationally validated with the purpose of identifying intimate partner violence, estimating frequency and severity, besides classifying subtypes of IPV^17,18^. It is self-completed by women, comprising four dimensions: emotional abuse, physical abuse, severe combined abuse, and harassment^19^. The instrument allows quantifying violence through a Likert-type scale, in which the woman answers how often abusive situations have occurred in the last twelve months (“never”, “once”, “sometimes”, “once a month”, “once a week” or “daily/almost daily”), generating at the end, a score of up to 150 points^17–19^. In the original instrument, the internal consistency reliability was 0.85 or above, for most subscales greater than 0.90, and the corrected item-total correlations were generally high (more than 0.5)^18^.

The CAS has been used to measure IPV in different contexts, including for case tracking/screening in health services, surveys, and online interventions^20–25^. It is considered the gold standard for measuring intimate partner violence^26^, with content, construct, criterion, and factor validity^18^. It has been translated from English into nine languages (Arabic, German, Spanish, Vietnamese, Dutch, Bengali, Russian, Japanese, and Malay)^19,27^, but not into Portuguese up to then.

Standardized IPV instruments allow comparability across different cultures, profiles and magnitudes of the problem. Other scales^28–31^ available in Brazilian Portuguese do not include all the types of intimate partner violence found in the CAS, nor do they generate scores to measure the frequency and severity of the problem. Therefore, we identified the gap in the CAS in Portuguese, and the need to adapt it to the Brazilian cultural context, aiming to measure IPV, its typologies, frequency and severity. The guiding question was: how to make available a version of the CAS that meets the idiomatic and cultural specificities of Brazil and at the same time is faithful to its original English version? This article discusses the translation and cross-cultural adaptation of the CAS into Brazilian Portuguese.

## METHODS

The protocol with the detailed methodology of the CAS translation and cross-cultural adaptation was previously published^32^, and rigorously followed, and the final results are described in this article. Briefly, the protocol contains ten steps (Figure): (a) conceptual analysis; (b) double-blind translation; (c) comparison and first reconciled version of the two translations (VR1); (d) back-translation; (e) developer's review of the back-translation and second reconciled version (VR2); (f) expert committee review; (g) comparison of expert reviews and third reconciled version (VR3); (h) cognitive interviews with users; (i) assessments of users’ perceptions and final reconciliation (VRF); and (j) presentation of the final version to the creator of the original instrument, in English^32^.

**Figure:**
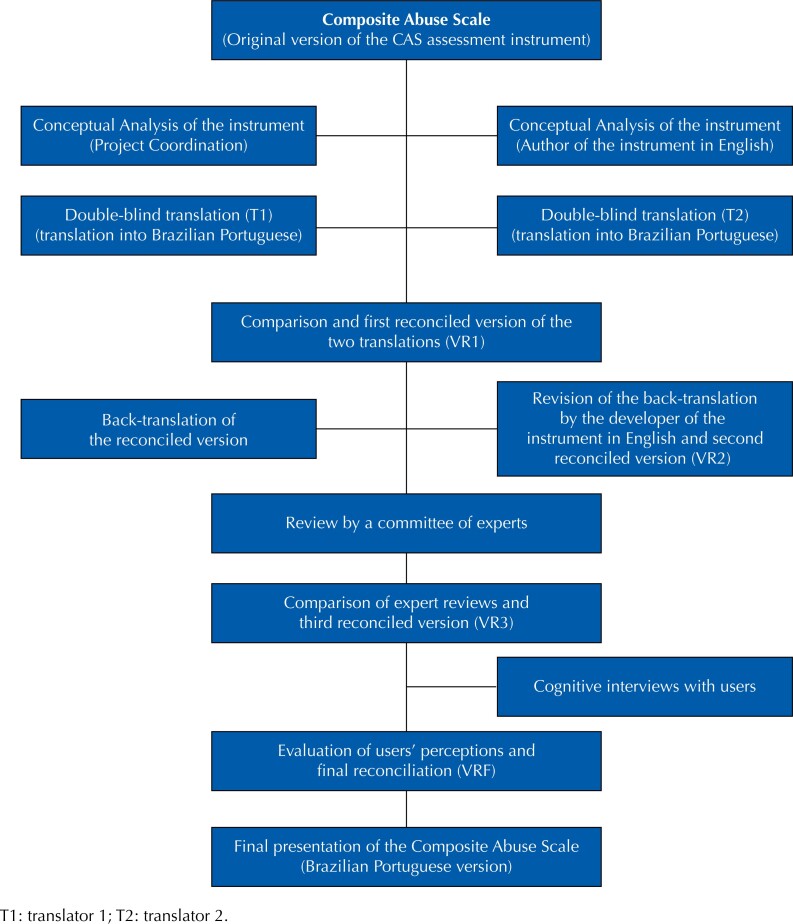
Flowchart of the translation and cross-cultural adaptation steps of the Composite Abuse Scale (CAS), from English into Brazilian Portuguese.

This paper is part of the first author's Masters in Collective Health. The developer of the original instrument actively participated in the process and is a co-author. Three translators with fluency in Portuguese and English (also co-authors) participated in steps (b) and (d), being: a PhD expert in gender-based violence; a Bachelor in translation, expert in translation and validation of health questionnaires; and a sworn translator.

To ensure the rigor of the translation and cross-cultural adaptation, invitations were sent to 12 experts in intimate partner and/or gender-based violence. The inclusion criteria for the experts were: Brazilian male and female researchers over 18 years old, with at least a doctoral degree, bound to universities or research centers, with research and scientific publications in the area of violence against women, and/or gender and violence studies. Of the 12 people invited, six agreed to participate in order to support the equivalences^33,34^: 1) semantic-idiomatic (the translation of the item keeps the meaning of the expression in the original version); 2) experiential (the situations evidenced in the items correspond to situations experienced in the Brazilian cultural context); and 3) conceptual (the situations presented in the items are easily understood by what is understood, in different populations of the country, about the concept addressed). The equivalence analysis by experts (Jan/2019-Feb/2019) occurred: quantitatively, comparing the English version with the VR2 and assigning a numerical score (0-100% equivalence); and qualitatively, through comments and suggestions for each item. The average numerical scores and suggestions are shown in Table 1. The number of experts was consistent with other translation and cross-cultural adaptation studies of IPV instruments^35–37^.

From June to September 2019, 15 cognitive interviews were conducted with women (users and professionals) from the *Casa da Mulher Brasileira* (House of the Brazilian Woman - CMB) of Curitiba. The CMB is an institution specialized in providing care to women experiencing violence, integrating all the services of the network in a single location^38^. The inclusion criteria for this stage were: i) professionals: over 18 years of age, who had been working at the CMB directly with women in situation of IPV for at least a year; ii) women: over 18 years of age, in situations of IPV and assisted by the CMB, who were not in crisis (as previously evaluated by the CMB professionals). Besides investigating what they understood about each item, they were also asked about the acceptability/applicability of the CAS to the Brazilian women. A printed version (VR3) was given to them to evaluate the layout/design of the instrument. The interviews lasted from 11 to 25 minutes, conducted in a private room of the CMB by an interviewer, a Master's student, occupational therapist, with a residency in women's health. Interviews were conducted until saturation was reached. After being recorded, they were transcribed and thematically analyzed according to each item of the instrument, following qualitative data analysis principles^39,40^.

At the end, the Brazilian version of the CAS was presented to the developer and the professionals at the CMB, being made available as an auxiliary tool in the assessment of IPV in women assisted there.

The study was approved by the Research Ethics Committees of the Federal University of Paraná (UFPR) and Curitiba City Hall, CAAE 89411818.4.000.0102. Participants signed the Informed Consent Form and were identified by codes, designated “E” (experts) and “P” (respondents). The study followed the WHO guidelines^41–43^ for research with women in violence. Only CMB users who were not in crisis, previously evaluated by the psychosocial department, participated.

## RESULTS

Chart 1 summarizes the process of translation and cross-cultural adaptation. In the first stage - conceptual analysis - the developer provided additional materials on the CAS development, including her thesis^44^ and the CAS handbook^19^. She also explained cultural differences found in translations into other languages, such as the Arabic translation, where one of the items (Item 25 - “Put foreign objects in my vagina”) did not match the standards of that culture, which would cause embarrassment to the respondents^27^.

**Chart 1:** Steps of the translation and cross-cultural adaptation of the Composite Abuse Scale into Brazilian Portuguese.

Original in English	Brazilian Portuguese version reconciled (VR1)	Portuguese version after review by the developer (VR2)	Experts’ comments and suggestions	Experts’ scoring	Cognitive Interviews with women from Casa da Mulher Brasileira	Final CAS version Brazilian Portuguese version
CAS Standard Version	*Versão Padrão EAC*	*Versão Padrão EAC*	ND	100%	ND	*CAS Versão Português Brasileiro*
Composite Abuse Scale (CAS) - Standard Version	*Escala de Abuso Composta (EAC) - Versão Padrão*	*Escala de Abuso Composta (EAC) - Versão Padrão*	ND	95.75%	ND	Composite Abuse Scale - *Versão Português Brasileiro*
Your relationships	*Seus relacionamentos*	*Seus relacionamentos*	ND	100%	ND	*Seus relacionamentos*
In this section we ask about your relationships because it is an important part of your life that may influence your health.	*Nesta seção, há perguntas sobre os seus relacionamentos, porque esta é uma parte importante de sua vida, que pode influenciar sua saúde.*	*Nesta seção, há perguntas sobre os seus relacionamentos, porque esta é uma parte importante de sua vida, que pode influenciar sua saúde.*	ND	100%	ND	*Nesta seção, há perguntas sobre os seus relacionamentos, porque esta é uma parte importante de sua vida, que pode influenciar sua saúde.*
We ask you about your experiences in adult intimate relationships.	*Perguntamos sobre as suas experiências em relações íntimas adultas.*	*Perguntamos sobre as suas experiências em relações íntimas adultas.*	“Why characterize as adult, and not simply ask after age 16? Adult sounds like mature. And this term we know is ambiguous. I think the word adult just gets in the way.” (E3) “We will ask about the behaviors of your affective/marital partners.” (E1's suggestion) “I have doubts whether the term ‘intimate relations’ accounts for a more refined understanding. Do intimate relations necessarily imply sexual intercourses? I think especially for couples who may not have had sexual intercourse yet.” (E2)	66.5%	“So, for me it is clear that it is a relation, a relationship, but many women will understand it as sexual intercourse, that you have had some intimate relations. Even because we use this term in a pejorative way, so as not to use the word sex, sexual…” (P7)	*Perguntamos sobre as suas experiências em relações afetivas ou conjugais.*
By adult intimate relationship we mean husband/wife, partner or boy/girl friend for longer than 1 month.	*Por relações íntimas adultas, nos referimos a esposo/esposa, parceiro/parceira ou namorado/namorada por um período maior que um mês.*	*Por relações íntimas adultas, nos referimos a esposo/esposa, parceiro/parceira ou namorado/namorada por um período maior que um mês.*	The term “intimate relation” is not part of the language of the great majority of the women who seek help, it can be misleading and/or depend on the interviewer to explain the term to the victim. That observation goes for all the very important issues, but that they use that term.” (E4) “Same question about the use of [previous] intimate relations.” (E2)	62.5%	“Oh, I think first of all…it's sex. Not necessarily an intimate relation as something that lasts for a period of time, right, you talked about a month, but I understand it as an act.” (P11)	*Por relações afetivas ou conjugais, nos referimos a esposo/esposa, parceiro/parceira ou namorado/namorada por um período maior que um mês.*
1. Have you ever been in an adult intimate relationship?	*1. Você já teve uma relação íntima adulta?*	*1. Você já teve uma relação íntima adulta?*	“Intimate relation is a dubious term; battered young women do not always have sexual activity, but this would not prevent them from answering the questions about violence. Even matching the answers could be another form of qualitative measurement, if some battered young women did not even have sexual relations with the abuser.” (E4)	66.5%	“[Intimate relation, to me is] sexual. The impression is that today we see that people are very concerned with the word sex. (…) So it is used, intimate, sometimes, even to…make it less invasive.” (P4)	*1. Você já teve um relacionamento afetivo ou conjugal?*
(Since you were 16 years of age)	*(Desde os seus 16 anos de idade)*	*(Desde os seus 16 anos de idade)*	ND	62.5%	ND	*(Desde os seus 16 anos de idade)*
( ) Yes 1 ( ) No 0 ( Go to next section)	*( ) Sim 1 ( ) Não 0 (Vá para a próxima seção)*	*( ) Sim 1 ( ) Não 0 (Vá para a próxima seção)*	ND	100%	ND	*( ) Sim 1 ( ) Não 0 (Vá para a próxima pergunta)*
2. Have you been in an adult intimate relationship in the last twelve months?	*2. Você teve alguma relação íntima adulta nos últimos doze meses?*	*2. Você teve alguma relação íntima adulta nos últimos doze meses?*	“Same question about the use of intimate relations”(E2). “It's always the problem with adult intimate relation” (E3). “The question is excellent, important, but the term ‘intimate relation’ complicates the answer…” (E4)	75%	“An intimate relation you can have with a non-partner person, it can be a person you met and it was just that intimate moment you had with them, not necessarily, you have some other contact in the future. More sexual involvement really.” (P8). “If I have had sex in the last 12 months?” (P3)	*2. Você teve algum relacionamento afetivo ou conjugal nos últimos doze meses?*
(Since you were 16 years of age)	*(Desde os seus 16 anos de idade)*	*(Desde os seus 16 anos de idade)*	ND	100%	ND	*(Desde os seus 16 anos de idade)*
( ) Yes 1 ( ) No 0 ( Go to question 6)	*( ) Sim 1 ( ) Não 0 ( Vá para a pergunta 6)*	*( ) Sim 1 ( ) Não 0 ( Vá para a pergunta 6)*	ND	87.5%	ND	*( ) Sim 1 ( ) Não 0 (Vá para a próxima pergunta)*
3. Are you currently in an intimate relationship?	*3. Você está em uma relação íntima adulta no momento?*	*3. Você está em uma relação íntima adulta no momento?*	“The question corresponds to English, but intimate relation sounds strange; wouldn't it be better to just ask: are you in a relationship now? That in Brazil everyone understands?” (E4)	79%	“For me it is a sexual intercourse (…) So, you understand that this question leads more to a sexual question?” (P15)	*3. Você está em um relacionamento afetivo ou conjugal no momento?*
( ) Yes 1 ( ) No 0 ( Go to question 5)	*( ) Sim 1 ( ) Não 0 ( Vá para a pergunta 5)*	*( ) Sim 1 ( ) Não 0 ( Vá para a pergunta 5)*	ND	100%	ND	*( ) Sim 1 ( ) Não 0 (Se não, por favor, vá para a pergunta 5)*
4. Are you currently afraid of your partner?	*4. Atualmente, você tem medo do seu parceiro ou parceira?*	*4. Atualmente, você tem medo do seu parceiro ou parceira?*	ND	95.75%	ND	*4. Atualmente, você tem medo do seu parceiro ou parceira?*
( ) Yes 1 ( ) No 0	*( ) Sim 1 ( ) Não 0*	*( ) Sim 1 ( ) Não 0*	ND	100%	ND	*( ) Sim 1 ( ) Não 0*
5. Have you been afraid of any partner in the last 12 months?	*5. Você teve medo do seu parceiro ou parceira nos últimos 12 meses?*	*5. Você teve medo do seu parceiro ou parceira nos últimos 12 meses?*	ND	79%	ND	*5. Você teve medo do seu parceiro ou parceira nos últimos 12 meses?*
( ) Yes 1 ( ) No 0	*( ) Sim 1 ( ) Não 0*	*( ) Sim 1 ( ) Não 0*	ND	50%	ND	*( ) Sim 1 ( ) Não 0*
6. Have you ever been afraid of any partner?	*6. Você já teve medo de algum parceiro ou parceira?*	*6. Você já teve medo de algum parceiro ou parceira?*	ND	79%	ND	*6. Você já teve medo de algum parceiro ou parceira?*
( ) Yes 1 ( ) No 0	*( ) Sim 1 ( ) Não 0*	*( ) Sim 1 ( ) Não 0*	ND	100%	ND	*( ) Sim 1 ( ) Não 0*
7. We would like to know if you experienced any of the actions listed below and how often it happened during the past twelve months. If you were not with a partner in the past twelve months, could you please answer for the last partner that you had. Please tick the appropriate box, which matches the frequency, over a twelve month period, that it happened to you.	*7. Gostaríamos de saber se você vivenciou alguma das ações listadas abaixo e com que frequência elas ocorreram nos últimos doze meses. Se você não teve um parceiro ou parceira nos últimos doze meses, por favor, responda à pergunta considerando o seu último parceiro ou parceira. Marque com um X a opção correta que corresponde à frequência com que a ação ocorreu com você nos últimos doze meses.*	*7. Gostaríamos de saber se você vivenciou alguma das ações listadas abaixo e com que frequência elas ocorreram nos últimos doze meses. Se você não teve um parceiro ou parceira nos últimos doze meses, por favor, responda à pergunta considerando o seu último parceiro ou parceira. Marque com um X a opção correta que corresponde à frequência com que a ação ocorreu com você nos últimos doze meses.*	ND	87.5%	ND	*7. Gostaríamos de saber se você vivenciou alguma das ações listadas abaixo e com que frequência elas ocorreram nos últimos doze meses. Se você não teve um parceiro ou parceira nos últimos doze meses, por favor, responda às perguntas considerando o seu último parceiro ou parceira. Marque com um X a opção correta que corresponde à frequência com que a ação ocorreu com você nos últimos doze meses.*
(Please tick one box on each line)	*(Por favor, marque uma das opções em cada linha)*	*(Por favor, marque uma das opções em cada linha)*	ND	83.25%	ND	*(Por favor, marque SOMENTE UMA das opções em cada linha)*
Actions	*Ações*	*Ações*	ND	87.5%	ND	*Ações*
How often it happened	*Com que frequência ocorreu*	*Com que frequência ocorreu*	ND	100%	ND	*Com que frequência ocorreu*
My Partner:	*Meu parceiro ou parceira:*	*Meu parceiro ou parceira:*	ND	100%	ND	*Meu parceiro ou parceira:*
Never	*Nunca*	*Nunca*	ND	100%	“I understood, zero who never did and even what is very frequent”. (P4)	*Nunca*
Only Once	*Somente uma vez*	*Uma vez*ᵃ	ND	100%	ND	*Uma vez*
Several Times	*Muitas vezes*	*Algumas vezes*ᵃ	ND	100%	ND	*Algumas vezes*
Once/Month	*Uma vez por mês*	*Uma vez por mês*	ND	100%	ND	*Uma vez por mês*
Once/Week	*Uma vez por semana*	*Uma vez por semana*	ND	100%	ND	*Uma vez por semana*
Daily	*Diariamente*	*Diariamente/Quase diariamente*ᵃ	ND	100%	ND	*Diariamente/Quase diariamente*
Told me that I wasn‟t good enough	*Falou que eu não era boa o suficiente*	*Falou que eu não era boa o suficiente*	ND	100%	ND	*Falou que eu não era boa o suficiente*
Kept me from medical care	*Impediu-me de obter tratamento médico*	*Impediu-me de obter tratamento médico*	“Change for colloquial language: for example, *me impediu*.” (E1)	100%	ND	*Me Impediu de obter tratamento médico*
Followed me	*Seguiu-me*	*Seguiu-me*	ND	87.5%	“I think that stalked me is better [than followed me]. From the moment I say: is stalking me, then he is bothering me, but followed me, it doesn't bother me, the word, you know, it doesn't bother me. It has no impact.” (P8)	*Me perseguiu*
Tried to turn my family, friends or children against me	*Tentou colocar minha família, amigos(as) ou filhos(as) contra mim*	*Tentou colocar minha família, amigos(as) ou filhos(as) contra mim*	ND	100%	ND	*Tentou colocar minha família, amigos(as) ou filhos(as) contra mim*
Locked me in the bedroom	*Trancou-me no quarto*	*Trancou-me no quarto*	ND	95.75%	ND	*Me trancou no quarto*
Slapped me	*Deu-me um tapa*	*Deu-me um tapa*	ND	100%	“As I told you, for the writing, for the spelling part it is correct, but the understanding, ‘me deu um tapa’ is stronger than saying ‘deu-me um tapa’.” (P8)	*Me deu um tapa*
Raped me	*Estuprou-me*	*Obrigou-me a ter relações sexuais contra a minha vontade*	ND	100%	I guess it is me *obrigou*. Know why? It [violence] has no social class. It takes all layers, from the simplest people to the most top, so… most culturally privileged, financially privileged, of privileged race, no color, no race, no social class.” (P14)	*Me obrigou a ter relações sexuais contra a minha vontade*
Told me that I was ugly	*Falou que eu era feia*	*Falou que eu era feia*	ND	100%	ND	*Falou que eu era feia*
Tried to keep me from seeing or talking to my family	*Tentou me impedir de ver ou falar com a minha família*	*Tentou me impedir de ver ou falar com a minha família*	ND	100%	ND	*Tentou me impedir de ver ou falar com a minha família*
Threw me	*Empurrou-me*	*Jogou-me e derrubou-me*	“Portuguese clearly corresponds to English; but if the instrument is intended to be comprehensive, colloquial Portuguese would be better; Example: *me impediu de ter tratamento médico; me trancou no quarto ou em casa; me obrigou a transar; me jogou e me derrubou no chão*. It may seem trivial, but the population doesn't understand formal Portuguese.” (E4)	91.5%	“*Jogou-me* and *derrubou-me*… is very strange… If you are talking about an assault, it's me *jogou* and me *derrubou*.” (P15)	*Me jogou e me derrubou*
Hung around outside my house	*Ficou me vigiando do lado de fora da minha casa*	*Ficou me vigiando do lado de fora da minha casa*	ND	95.75%	ND	*Ficou me vigiando do lado de fora da minha casa*
Blamed me for causing their violent behaviour	*Culpou-me por ter causado seu comportamento violento*	*Culpou-me por ter causado seu comportamento violento*	ND	100%	ND	*Me culpou por ter causado seu comportamento violento*
Harassed me over the telephone	*Assediou-me pelo telefone*	*Assediou-me pelo telefone, internet ou redes sociais*ᵃ	ND	95.75%	ND	*Me assediou pelo telefone, internet ou redes sociais*
Shook me	*Chacoalhou-me*	*Chacoalhou-me*	ND	95.75%	“Look, there's a very strange word: *chacoalhou*, *chacoalhou* what? *Pegou e me chacoalhou*… *Me sacudiu* I think it's better.” (P9)	*Me sacudiu*
Tried to rape me	*Tentou me estuprar*	*Tentou me forçar a ter relações sexuais contra a minha vontade*	ND	100%	ND	*Tentou me forçar a ter relações sexuais contra a minha vontade*
Harassed me at work	*Assediou-me no trabalho*	*Assediou-me no trabalho*	ND	100%	ND	*Me assediou no trabalho*
Pushed, grabbed or shoved me	*Empurrou-me ou agarrou-me*	*Empurrou-me ou agarrou-me*	ND	100%	ND	*Me empurrou ou me agarrou*
Used a knife or gun or other weapon	*Usou uma faca, um revólver ou outra arma*	*Usou uma faca, um revólver ou outra arma contra mim*	ND	100%	ND	*Usou uma faca, um revólver ou outra arma contra mim*
Became upset if dinner/housework was not done when they thought it should be.	*Ficou bravo/brava se o jantar ou afazer doméstico não foi feito do modo que ele(a) achava que deveria*	*Ficou bravo/brava se o jantar ou afazer doméstico não foi feito do modo que ele(a) achava que deveria*	ND	91.5%	ND	*Ficou bravo/brava se o jantar ou afazer doméstico não foi feito do modo que ele/ela achava que deveria*
Told me that I was crazy	*Disse-me que era louca*	*Disse-me que era louca*	ND	100%	ND	*Me disse que era louca*
Told me that no one would ever want me	*Disse-me que ninguém nunca vai me querer*	*Disse-me que ninguém nunca vai me querer*	ND	100%	ND	*Me disse que ninguém nunca vai me querer*
Took my wallet and left me stranded	*Pegou a minha carteira e deixou-me sem dinheiro*	*Pegou a minha carteira e deixou-me sem dinheiro*	ND	95.75%	ND	*Pegou a minha carteira e me deixou sem dinheiro*
Hit or tried to hit me with something	*Bateu ou tentou me bater com alguma coisa*	*Bateu ou tentou me bater com alguma coisa*	ND	95.75%	ND	*Bateu ou tentou me bater com alguma coisa*
Did not want me to socialise with my female friends	*Não quis que eu me encontrasse com minhas amigas*	*Não quis que eu me encontrasse com minhas amigas*	“In the Brazilian Portuguese version post-revision by the author is equivalent to the original English language version. However, it is not equivalent to the Brazilian Portuguese version because it does not maintain the meaning of the expression. In this specific case, I believe that from the point of view of women's experiences, the addition of the word “*amigos*” in the Brazilian Portuguese version contributes to the evidence (or not) of the occurrence of the IPV phenomenon (which meets the “experimental” relevance item). (E5)	95.75%	ND	*Não quis que eu me encontrasse com minhas amigas/amigos*
Put foreign objects in my vagina	*Colocou objetos estranhos na minha vagina*	*Colocou objetos estranhos na minha vagina contra a minha vontade*	ND	100%	ND	*Colocou objetos estranhos na minha vagina contra a minha vontade*
Refused to let me work outside the home	*Não deixou que eu trabalhasse fora de casa*	*Não deixou que eu trabalhasse fora de casa*	ND	100%	ND	*Não deixou que eu trabalhasse fora de casa*
Kicked me, bit me or hit me with a fist	*Chutou-me, mordeu-me ou me deu socos*	*Chutou-me, mordeu-me ou me deu socos*	ND	100%	ND	*Me chutou, me mordeu ou me deu socos*
Tried to convince my friends, family or children that I was crazy	*Tentou convencer meus amigos, família ou filhos(as) que eu era louca*	*Tentou convencer meus amigos, família ou filhos(as) que eu era louca*	ND	100%	ND	*Tentou convencer meus amigos(as), família ou filhos(as) que eu era louca*
Told me that I was stupid	*Disse-me que era burra*	*Disse-me que era burra*	ND	100%	ND	*Me disse que eu era burra*
Beat me up	*Bateu em mim*	*Bateu em mim*	ND	100%	ND	*Bateu em mim*

ND: nothing to declare; IPV: intimate partner violence; E1,E2…: expert 1, expert 2…; P3, P4…: respondent 3, respondent 4…

a The alternatives were suggested by the developer of the instrument based on the 2016 revision of the Composite Abuse Scale (Ford-Gilboe et al.26, 2016)^26^.

In step (b), some divergences between the two translators (T1 and T2) were discussed between the research team (masters student and advisors) in meetings for comparison, analysis, and summary between the two translated versions and the original instrument. Translators have also contributed to clarify doubts, thus obtaining the VR1 (c), presented in Chart 1.

In step (d) VR1 was back-translated in a blinded way, without the back-translator knowing the original instrument, nor communicating with the translators. The back-translator sent some questions to the research coordinating team about words that had no literal translation in Portuguese, but had a different connotation of intensity in the English language (e.g. item 17 - “Pushed, grabbed or shoved me”) or gender neutrality in the English instrument (e.g. “partner”), which implies a gender inflection in Portuguese (“parceiro”/”parceira”).

The back-translated version was sent to the developer for analysis (step e). Doubts arising in the previous steps were also pointed out to the developer, in a meeting to seek consensus. The developer's opinion was crucial to maintain the reliability of the original instrument, but at the same time keeping the language updated and contextualized to the Brazilian scenario. One of the adjustments at this stage included the adaptation of item 7, “Raped me” for “Forced me to have sex against my will”, since many married women (or in a relation) do not perceive forced sexual intercourses within marriage/relationship as rape. Another adaptation was in item 25 (“Put foreign objects in my vagina”) which became “Put foreign objects in my vagina against my will” to differentiate from consensual relations. The developer also suggested updating some items based on a recent revision of CAS^26^, as in item 13, “Harassed me over the telephone”, which now also includes Internet and social media. At the end of step (e), we got to VR2, detailed in Chart 1.

Six specialists (S), professors at universities in different regions of the country, whose profiles are shown in Table 2, participated in step (f). The areas of training were quite diverse, including Health, Psychology, Human and Exact Sciences, enabling multidisciplinary views on the translation and cross-cultural adaptation of the CAS.

**Chart 2:** Profile of experts (E) participating in the expert analysis step (step f).

Expert	Gender	Schooling	Highest degree
E1	Woman	Nursing and Philosophy	Post-Doctorate in Technology
E2	Woman	Sciences with Major in Mathematics	Postdoctoral Fellow in Interdisciplinary Studies on Women, Gender and Feminisms
E3	Man	Psychology	PhD in Collective Health
E4	Woman	Medicine	PhD in Preventive Medicine
E5	Woman	Social and Political Sciences	PhD in Interdisciplinary Humanities
E6	Woman	Psychology	PhD in Collective Health

E1, E2…, E6: Expert 1, Expert 2…, Expert 6.

Four out of six experts problematized the term “adult intimate relations” and why we did not use “sexual intercourses” or “affective-sexual relations”. Regarding the term “adult intimate relations”, the instrument itself explains its meaning, as can be seen in Table 1 (VR2): “By adult intimate relations, we refer to spouse, male or female partner, or boyfriend/girlfriend for a period longer than one month”. The committee drew attention to the fact that in Brazil, unlike in other countries, 16-year-old women are not considered adults, since the instrument can be completed by people as young as 16. Thus, we removed the word “adult”, but kept the term “intimate relations” because of the explanation of this term, which comes right after in the instrument.

Experts have also showed concern with the understanding of the items and commands for proper completion, reinforcing that the language should be more colloquial than academic. They signaled the need to exchange enclisis for proclisis cases (e.g.: “me derrubou” instead of “derrubou-me”); and need for gender inflection, as in item 24 - “Did not want me to socialise with my female friends” for “Did not want me to meet with my female/male friends”, since it is common in Brazilian society for women to also have male friends.

Based on the experts’ considerations and conciliation meetings among the team, we reached VR3, which was then submitted to the cognitive interviews with women from the CMB, in order to assess whether the items were understood as expected. Of the 15 participants in this stage, seven identified themselves as white, four as black, and four did not know; 11 identified themselves as heterosexual, two as bisexual, and two as lesbian, all of them cis women. As for income, three participants had family incomes of up to one minimum wage, two between one and two minimum wages, one between two and three minimum wages, and nine had three or more minimum wages. Eight participants were the main responsible for the family income. As for education, two had a high school degree, four had a college degree, and nine had a graduate degree.

After providing a hardcopy (VR3), we asked them what they understood by each item. Some doubts emerged, shown in Chart 1 (column “Cognitive interviews”). The most problematic term was “intimate relation”, which had already been problematized in the expert stage. The women's understanding of intimate relations generally disagreed with the original concept, stating that “intimate relation” was understood as “sexual intercourse”. The interviewer noticed that even after reading the explanatory excerpt from the CAS describing what is considered an intimate relation, participants continued to associate the term with sexual intercourse. Even the participants who, after reading the concept, understood it as referring to a dating, marriage, or intimate partner relation, throughout the interview again associated the intimate relation with sexual intercourse. This interpretation occurred in all the questions that contained this term. Participant P3, when asked about her understanding of the question “Have you had any intimate relations in the last twelve months?”, immediately responded: “*Do you want to know if I have had sex in the last 12 months?*”

The participants’ criticisms converged with the experts’ notes and, considering their suggestions, the interviewer inquired if the question should be rephrased to “Have you had any affective or conjugal relations…?” Participants then understood that yes, the question would be related to dating, marriage, or affective partnership. They also unanimously agreed that the new wording improved understanding, as exemplified by P4: “*In this question it's in a, like, different tone, you know, I can't see the sexual aspect like in the others. I can see it as something like, there's a dating”.*

Another question raised by participants was about the language of the instrument and its experiential equivalence. Most recommended a more colloquial language, without the use of the enclisis case, as already pointed out by the experts. P4 exemplifies: “…*slapped me, it's popular language…it's the language that most people use*…” When P15 was asked if it would be more intelligible “*Me jogou e me derrubou*” instead of “*Jogou-me e derrubou-me*” (Threw me and knocked me down), the participant replied: *“If you're talking about an assault it's…me jogou e me derrubou.”* Based on this, all the enclisis cases were replaced by proclisis cases, as stated in the final version of the instrument, aware that they do not correspond to the correct grammar of the Portuguese language, but paying attention to the needs of understanding and interpretation by the population at large.

Some participants also challenged item 3 (“Followed me”), because they considered that it did not convey the idea of violence or could generate double meaning, thinking in the context of social media (“followed me on Facebook”). Some suggested that “stalked me” would be more appropriate. Even those who did not suggest changing the term, when explaining what they understood about it they used the word “stalk”: “*The partner stalked me*” (P10); “*Someone is stalking me*” (P11). The item was then changed to “Stalked me”.

In item 14 (“*Chacoalhou-me*”), 11 out of 15 participants felt that it would be better to replace it with “*Me sacudiu*” (shook me). Even though they understood the meaning of the term “*Chacoalhou-me*”, the word “*Sacudir*” would sound better, as exemplified by (P6): “*I think ‘me sacudiu’ sounds better…*”. P14 adds that: “‘*Me sacudiu*’, I think anyone can get it”. She considers, therefore, more accessible to the understanding of people with different schooling levels.

In the other items, there were no discordant notes, with a consensual understanding. Some participants highlighted the instrument's relevance for the Brazilian women, saying that the CAS is compatible with the Brazilian scenario, it is practical, useful, and may even be necessary. One of the professionals’ comments in this sense:

*When they get here [CMB], this whole thing here, well I mean, it has already occurred. Many of these [situations] have already occurred. And she really doesn't perceive it. And from the moment that she responds to this here, she will say: My God! This is what happens to me.* (P8)

She, then, supports the compatibility of the CAS with the reality of the women who seek the CMB, and that the instrument can even help them to visualize and understand the situations of violence to which they are subjected in their relationships.

Some items drew the researchers’ attention, such as “2 - Prevented me from getting medical treatment”, according to P5, “*This is very common, partners don't allow them to attend or follow up*”. This signals challenges for the health field in caring for these women considering comments from professionals who work every day with women in situations of violence. Thus, the research team considers this a relevant finding for the compatibility of the instrument with the Brazilian cultural context, even though it was created in another country.

As for the design of the instrument and completion of the items, some participants praised it for its objectivity and ease of understanding, such as P6: “*I think it looks great like that. Much better than those evaluations, scales, which I think are terrible, choose partially, partially agree. Here it is very clear and objective*”. The participant refers to some instruments that use Likert scales^45^ as a method of completing. On the other hand, two participants made criticisms of the instrument's design, which should be incorporated into the final version. These were the need for greater spacing between items to facilitate visualization, as well as the use of lines or different shading to separate one question from another.

## DISCUSSION

The implementation of the protocol^32^ for translation and cross-cultural adaptation of the CAS resulted in the “CAS Brazilian Portuguese Version”, adapted to the context of the Brazilian women and with idiomatic, semantic, conceptual, and experiential equivalence^33,34^. The instrument may be self-applied, generating a score that allows classifying the woman as living in a situation of intimate partner violence (score ≥ 7 points), in a score range that varies from 0 to 150 points^18,19^. The field research highlighted that the scale may help women visualize situations of abuse experienced in their relationships, helping them to perceive IPV.

The main adaptations made throughout the process consisted of changing some terms, such as replacing “intimate relations” for “affective or conjugal relationships” because they have a better equivalence to the original construct, which refers to affective relationships with partners of at least one month duration, and not only sexual intercourses. A meaningful adaptation was the exchange of enclisis for proclisis cases in 20 items, aware that this change does not contemplate the grammar according to the Portuguese language norms, but meets a demand of the potential users, facilitating their understanding. Another significant adaptation was the incorporation of neutral language, such as partner, allowing the instrument to be used by women in heterosexual, bisexual, or same-sex relationships.

Another aspect was the replacement of the term “Rape me” for “Forced me to have sex against my will”, taking into account findings in literature showing that women do not believe they can be raped within affective/conjugal relationships and, therefore, do not associate this type of sexual relation with intimate partner violence^46–48^. Similarly, we also adapted item 15 “Tried to rape me” to “Tried to force me to have sex against my will”.

Additional questions problematized by the expert committee related to the types of relationships, suggesting to include other intimate bonds and practices (such as polyamory, for example), as well as friendship relationships. However, concerned to maintain the reliability of the original instrument, the research team believes that the CAS presents this limitation for not including these other types of relationships. However, this is a gap that cannot be solved in this study, being necessary another type of research or the creation of a new instrument that specifically addresses these issues. Even considering as important to think over the many types of bonds existing in intimate relations, one of the purposes of the translation and cross-cultural adaptation of a scale into another language/culture is the possibility of comparing the results obtained from its application in other countries/languages/cultures. Therefore, the instrument cannot undergo substantial changes that would not allow comparability of responses between languages. In the case of the CAS, instructions are explicit with regard to the instrument completion, which should consider the person who perpetrated the violence and who has been for at least one month in a relationship (affective or conjugal) with the person who is completing the scale.

The cognitive interview stage was crucial in the translation and cross-cultural adaptation process to investigate the understanding of the women who will be the potential users of the CAS. Collins^49^ explains that the questions in this step, particularly when applied in the context of the instrument, allow us to determine whether respondents can understand the concept or task of the question, and whether they do so consistently and in the manner intended by the researcher. Thus, conducting this step at CMB which exclusively serves women in situations of violence, proved to be an effective strategy for adapting the CAS to the needs of the Brazilian women. The content, meanings, and presentation format of the instrument were evaluated by potential users and by the professionals who assist them daily.

Regarding the layout, even though only two of the 15 participants in this stage criticized the design, one can see in their statements the importance of absorbing the criticism to make the instrument completion as intuitive and easy as possible, since the CAS was designed to be self-completed by women.

The study had as main limitations the fact that cognitive interviews were conducted in one single place, in the Southern Region of Brazil, and with no participation of women with low education. Women with a lower level of education may have some difficulty in completing the questionnaire, and regional idiomatic nuances from other areas of the country may not have been sufficiently incorporated into the instrument. Therefore, it is recommended in future studies that the Brazilian CAS be applied to a larger number of women, with more diversified education levels. On the other hand, the stage with experts included researchers from different regions of the country, who were invited to carry out their analysis having in mind the diverse public of Brazilian women (regionalisms, levels of education, age group, etc.). And, in fact, notes were made in this direction, such as the simplification of the language to facilitate the respondents’ understanding.

In brief, participants gave a verdict of pertinence to the CAS Brazilian Portuguese Version. Besides the positive evaluation regarding linguistic comprehension, for them the instrument incorporated aspects about intimate partner violence that also concern the Brazilian reality and, therefore, should be monitored. Thus, the process of translation and cross-cultural adaptation was successfully completed, and the CAS Brazilian Portuguese version is available for use by the Brazilian academic community. It is worth pointing out the need for the instrument to have its psychometric properties evaluated in future studies. Thus, it may be made available according to the highest methodological standards and scientific rigor for wide use in Brazil.
