# Supplementary material for: Translation and cross-cultural adaptation of the Composite Abuse Scale into Brazilian Portuguese
Source: Rev Saude Publica. 2022 Nov 18;56:98. doi: 10.11606/s1518-8787.2022056004196 (PMC9749724; doi:10.11606/s1518-8787.2022056004196)
Supplement: Translation

# Translation: Tradução e adaptação transcultural da Composite Abuse Scale para o português brasileiro

**Authors:** Raiza Wallace Guimaraes da Rocha, Daniel Canavese de Oliveira, Vitor Adriano Liebel, Patricia Helena Rubens Pallu, Kelsey Lee Hegarty, Marcos Claudio Signorelli

**Affiliations:** I Curitiba PR Brasil Universidade Federal do Paraná. Programa de Pós-Graduação em Saúde Coletiva. Curitiba, PR, Brasil; II Porto Alegre RS Brasil Universidade Federal do Rio Grande do Sul. Departamento de Saúde Coletiva. Porto Alegre, RS, Brasil; III Curitiba PR Brasil V.A. Liebel Linguistic Solutions. Curitiba, PR, Brasil; IV Curitiba PR Brasil Colégio Positivo Junior. Curitiba, PR, Brasil; V Melbourne VIC Australia University of Melbourne. Department of General Practice. The Royal Women's Hospital. Melbourne, VIC, Australia; VI Curitiba PR Brasil Universidade Federal do Paraná. Setor de Ciências da Saúde. Departamento de Saúde Coletiva. Curitiba, PR, Brasil

**Keywords:** Violência contra a Mulher, Violência por Parceiro Íntimo, Inquéritos e Questionários, Tradução, Comparação Transcultural

## Abstract

**OBJETIVO:**

Realizar a tradução e adaptação transcultural do inglês para o português brasileiro da *Composite Abuse Scale*, instrumento que identifica e quantifica a violência por parceiro íntimo.

**MÉTODOS:**

Este estudo baseia-se na execução rigorosa de seu protocolo, previamente publicado, e que consiste em dez etapas: (a) análise conceitual; (b) tradução duplo-cega; (c) comparação e primeira versão reconciliada das duas traduções; (d) retrotradução; (e) revisão da retrotradução feita pela desenvolvedora e segunda versão reconciliada; (f) revisão por comitê de especialistas (n = 6); (g) comparação das revisões por especialistas e terceira versão reconciliada; (h) entrevistas cognitivas com mulheres da Casa da Mulher Brasileira de Curitiba (n = 15); (i) avaliações das percepções das usuárias e reconciliação final; e (j) apresentação da versão final do questionário à desenvolvedora.

**RESULTADOS:**

A execução das 10 etapas do protocolo permitiu as equivalências idiomática, semântica, conceitual e experiencial da *Composite Abuse Scale*, incorporando sugestões e críticas dos diferentes participantes do processo, que incluíram desde a desenvolvedora, tradutores profissionais, pesquisadores especializados no tema, mulheres em situação de violência por parceiro íntimo e profissionais que as atendem. Especialistas e entrevistas cognitivas com mulheres foram fundamentais para garantir equivalências e facilitar a compreensão, incluindo: (1) adaptação do termo “relacionamento íntimo” para “relacionamento afetivo ou conjugal”; (2) substituição de ênclises por próclises em 20 itens; (3) adoção de linguagem de gênero neutro, permitindo sua utilização em relacionamentos hetero, bi e homoafetivos; (4) materialização de um instrumento de rigor científico e autoaplicável, que pode auxiliar as mulheres a visibilizarem as situações de abuso em seus relacionamentos.

**CONCLUSÕES:**

O processo de tradução e adaptação transcultural da *Composite Abuse Scale* resultou na *Composite Abuse Scale* Versão Português brasileiro, instrumento autoaplicável com 30 itens, capaz de identificar e quantificar a violência por parceiro íntimo, sua frequência, severidade e tipologias (violências física, emocional, assédio e grave combinada).

## INTRODUÇÃO

A violência por parceiro íntimo (VPI) é um problema global de saúde pública^1,2^. Estimativas da Organização Mundial da Saúde (OMS) apontam que 23% das mulheres brasileiras já sofreram VPI^3^. Entre 2011 e 2017, a maioria das notificações de violência de gênero no Sistema Único de Saúde (SUS) foi por violência por parceiro íntimo^4–6^.

A eliminação da VPI é um dos objetivos do desenvolvimento sustentável para 2030^7^, do qual o Brasil é signatário. O país tem envidado esforços^8,9^ para prevenir esse tipo de violência, contudo, desafios persistem^10^. Em 2021 foi aprovada legislação específica para tipificar a violência psicológica contra as mulheres^11^, estimado como o subtipo mais prevalente de violência por parceiro íntimo^12^, porém é a violência mais difícil de se identificar quando comparada aos outros subtipos de VPI (física ou sexual) e é descrita como a mais impactante para a saúde das mulheres^12,13^. Portanto, é necessário que existam instrumentos de confiabilidade^14,15^ para identificação e mensuração desse tipo de problema (violência psicológica), sem desconsiderar os demais subtipos de VPI.

Identificar e quantificar a VPI e seus subtipos, utilizando-se métodos comparáveis e cientificamente rigorosos contribui para o monitoramento e o subsídio à tomada de decisões^16^. Nessa direção, a *Composite Abuse Scale* (CAS) é uma escala desenvolvida na Austrália, validada internacionalmente com o intuito de identificar a violência por parceiro íntimo, estimar frequência e severidade, além de classificar subtipos de VPI^17,18^. É autopreenchida pelas mulheres, contemplando quatro dimensões: abuso emocional, abuso físico, abuso combinado grave e assédio^19^. O instrumento permite quantificar a violência por meio de uma escala do tipo Likert, em que a mulher responde com que frequência situações abusivas ocorreram nos últimos doze meses (“nunca”, “uma vez”, “algumas vezes”, “uma vez por mês”, “uma vez por semana” ou “diariamente/quase diariamente”), gerando ao final, um escore de até 150 pontos^17–19^. No instrumento original, a confiabilidade da consistência interna foi de 0,85 ou acima, para a maioria das subescalas maior que 0,90 e as correlações item-total corrigidas foram geralmente altas (mais de 0,5)^18^.

A CAS tem sido utilizada para mensurar a VPI em diferentes contextos, inclusive para rastreamento/triagem de casos em serviços de saúde, *surveys* e intervenções online^20–25^. É considerada padrão ouro para mensurar violência por parceiro íntimo^26^, com validade de conteúdo, construto, critério e fator^18^, traduzida do inglês para nove idiomas (árabe, alemão, espanhol, vietnamita, holandês, bengali, russo, japonês e malaio)^19,27^, mas, até então, não fora traduzida para o português.

Instrumentos padronizados de VPI permitem comparabilidade entre diferentes culturas, perfis e magnitudes do problema. Outras escalas^28–31^ disponíveis em português brasileiro não contemplam todas as tipologias de violência por parceiro íntimo encontradas na CAS, nem geram escore para dimensionar frequência e severidade do problema. Portanto, identificou-se a lacuna da CAS em português e a necessidade de adaptá-la ao contexto cultural brasileiro, objetivando mensuração de VPI, suas tipologias, frequência e severidade. A questão norteadora foi: como disponibilizar uma versão da CAS que atenda às especificidades idiomáticas e culturais do Brasil e ao mesmo tempo fiel à sua versão original em inglês? O presente artigo discorre sobre a tradução e adaptação transcultural da CAS para o português brasileiro.

## MÉTODOS

O protocolo com a metodologia detalhada da tradução e adaptação transcultural da CAS foi publicado previamente^32^, rigorosamente seguido e os resultados finais são descritos neste artigo. Resumidamente, o protocolo contém dez etapas (Figura): (a) análise conceitual; (b) tradução duplo-cega; (c) comparação e primeira versão reconciliada das duas traduções (VR1); (d) retrotradução; (e) revisão da retrotradução pela desenvolvedora e segunda versão reconciliada (VR2); (f) revisão por comitê de especialistas; (g) comparação das revisões por especialistas e terceira versão reconciliada (VR3); (h) entrevistas cognitivas com usuárias; (i) avaliações das percepções das usuárias e reconciliação final (VRF); e (j) apresentação da versão final à criadora do instrumento original, em inglês^32^.

**Figura:** Fluxograma das etapas de tradução e adaptação transcultural da *Composite Abuse Scale* (CAS), do inglês para o português brasileiro.

Este trabalho é parte do mestrado em Saúde Coletiva da primeira autora. A desenvolvedora do instrumento original participou ativamente do processo e é coautora. Três tradutores com fluência em português e inglês (também coautores) participaram das etapas (b) e (d), sendo: um doutor, especialista em violência de gênero; um bacharel em tradução, especialista em tradução e validação de questionários de saúde; e uma linguista tradutora juramentada.

Para garantir o rigor da tradução e adaptação transcultural foram enviados convites a 12 especialistas em violência por parceiro íntimo e/ou gênero. Os critérios de inclusão de especialistas foram: pesquisadores e pesquisadoras brasileiros maiores de 18 anos, com titulação mínima de doutorado, vinculados a universidades ou centros de pesquisa, com pesquisas e publicações científicas na área de violência contra mulheres e/ou estudos de gênero e violência. Dos 12 convidados, seis aceitaram participar para apoiar as equivalências^33,34^: 1) semântico-idiomática (a tradução do item mantém o sentido da expressão na versão original); 2) experiencial (as situações evidenciadas nos itens correspondem às situações vivenciadas no contexto cultural brasileiro); e 3) conceitual (as situações apresentadas nos itens são de fácil compreensão pelo que se entende, em diferentes populações do país, sobre o conceito abordado). A análise de equivalências por especialistas (jan/2019-fev/2019) ocorreu: quantitativamente, comparando a versão em inglês com a VR2 e atribuindo um escore numérico (0–100% de equivalência); e qualitativamente, por meio de comentários e sugestões para cada item. A média dos escores numéricos e sugestões constam no Quadro 1. O número de especialistas foi condizente com outros estudos de tradução e adaptação transcultural de instrumentos de VPI^35–37^.

Entre junho e setembro 2019 foram conduzidas 15 entrevistas cognitivas com mulheres (usuárias e profissionais) da Casa da Mulher Brasileira (CMB) de Curitiba, instituição especializada no atendimento às mulheres experienciando violência, integrando em único local todos os serviços da rede^38^. Os critérios de inclusão para essa etapa foram: i) profissionais: maiores de 18 anos, e que trabalhassem na CMB, diretamente no atendimento às mulheres em situação de VPI há pelo menos um ano; ii) mulheres: maiores de 18 anos, em situação de VPI e atendidas pela CMB, que não estivessem em crise (conforme avaliação prévia feita pelas profissionais da CMB). Além de investigar o que entendiam sobre cada item, também foram perguntados aspectos relativos à aceitabilidade/aplicabilidade da CAS às brasileiras. Foi entregue uma versão impressa (VR3) para que avaliassem o layout/design do instrumento. As entrevistas tiveram duração de 11 a 25 minutos, conduzidas em sala privativa da CMB, por entrevistadora mestranda, terapeuta ocupacional, com residência em saúde da mulher. As entrevistas foram conduzidas até atingir a saturação. Após gravadas, foram transcritas e analisadas tematicamente de acordo com cada item do instrumento, seguindo princípios da análise de dados qualitativos^39,40^.

Ao final, a versão brasileira da CAS foi apresentada à desenvolvedora e às profissionais da CMB, sendo disponibilizada como instrumento auxiliar na avaliação de VPI das mulheres lá atendidas.

O estudo foi aprovado pelos Comitês de ética em esquisas da Universidade Federal do Paraná (UFPR) e Prefeitura de Curitiba, CAAE 89411818.4.000.0102. As participantes assinaram o termo de consentimento e foram identificadas por códigos, denominadas “E” (especialistas) e “P” (entrevistadas). O estudo seguiu as diretrizes da OMS^41–43^ para pesquisas com mulheres em violência. Somente participaram usuárias da CMB que não estavam em crise, previamente avaliadas pelo departamento psicossocial.

## RESULTADOS

O Quadro 1 sintetiza o processo de tradução e adaptação transcultural. Na primeira etapa – análise conceitual – a desenvolvedora forneceu materiais adicionais do desenvolvimento da CAS, incluindo sua tese^44^ e manual da CAS^19^. Também explicou diferenças culturais encontradas em traduções para outros idiomas, como exemplo a tradução para o árabe, em que um dos itens (Item 25 – “*Put foreign objects in my vagina*”) não condizia aos padrões daquela cultura, o que causaria constrangimentos às respondentes^27^.

**Quadro 1:** Etapas da tradução e adaptação transcultural da *Composite Abuse Scale* para o português brasileiro.

Original em inglês	Versão em português brasileiro reconciliada (VR1)	Versão em português após revisão da desenvolvedora (VR2)	Comentários e sugestões das(os) especialistas	Escore dos(as) especialistas	Entrevistas Cognitivas com mulheres da Casa da Mulher Brasileira	Versão final da CAS Versão Português Brasileiro
*CAS Standard Version*	Versão Padrão EAC	Versão Padrão EAC	ND	100%	ND	CAS Versão Português Brasileiro
*Composite Abuse Scale (CAS) - Standard Version*	Escala de Abuso Composta (EAC) - Versão Padrão	Escala de Abuso Composta (EAC) - Versão Padrão	ND	95,75%	ND	*Composite Abuse Scale* - Versão Português Brasileiro
*Your relationships*	Seus relacionamentos	Seus relacionamentos	ND	100%	ND	Seus relacionamentos
*In this section we ask about your relationships because it is an important part of your life that may influence your health.*	Nesta seção, há perguntas sobre os seus relacionamentos, porque esta é uma parte importante de sua vida, que pode influenciar sua saúde.	Nesta seção, há perguntas sobre os seus relacionamentos, porque esta é uma parte importante de sua vida, que pode influenciar sua saúde.	ND	100%	ND	Nesta seção, há perguntas sobre os seus relacionamentos, porque esta é uma parte importante de sua vida, que pode influenciar sua saúde.
*We ask you about your experiences in adult intimate relationships.*	Perguntamos sobre as suas experiências em relações íntimas adultas.	Perguntamos sobre as suas experiências em relações íntimas adultas.	“Por que caracterizar como adulta, e não perguntar simplesmente depois dos 16 anos? Adulto soa como madura. E este termo sabemos é ambíguo. Acho que a palavra adulta só atrapalha.” (E3) “Perguntaremos sobre os comportamentos de seus/suas parceiros/as afetivas/conjugais.” (Sugestão da E1) “Tenho dúvidas se o termo “relações íntimas” dão conta de um entendimento mais apurado. Relações íntimas implicam necessariamente em relações sexuais? Penso sobretudo para os namorados que porventura ainda não tenham tido relações sexuais.” (E2)	66,5%	“Então, pra mim fica claro, que é uma relação, é um relacionamento, né, mas muitas mulheres vão entender que é uma relação sexual, que você teve alguma relação íntima. Até porque a gente usa esse termo, de forma pejorativa, né, assim, pra não falar a palavra sexo, sexual, enfim…” (P7)	Perguntamos sobre as suas experiências em relações afetivas ou conjugais.
*By adult intimate relationship we mean husband/wife, partner or boy/girl friend for longer than 1 month.*	Por relações íntimas adultas, nos referimos a esposo/esposa, parceiro/parceira ou namorado/namorada por um período maior que um mês.	Por relações íntimas adultas, nos referimos a esposo/esposa, parceiro/parceira ou namorado/namorada por um período maior que um mês.	“O termo relações íntimas não faz parte da linguagem da grande maioria das mulheres que procura ajuda, pode induzir a erro e/ou depender de entrevistador/a para explicar o termo para a pessoa agredida. Essa observação serve para todas as importantíssimas questões, mas que utilizam esse termo.” (E4) “Mesma questão sobre o uso de relações íntimas [anterior].” (E2)	62,5%	“Ah, eu penso em primeiro lugar… é sexo. Não necessariamente uma relação íntima como algo que tenha um período que dura, né, você falou num mês, mas eu entendo como um ato.” (P11)	Por relações afetivas ou conjugais, nos referimos a esposo/esposa, parceiro/parceira ou namorado/namorada por um período maior que um mês.
*1. Have you ever been in an adult intimate relationship?*	1. Você já teve uma relação íntima adulta?	1. Você já teve uma relação íntima adulta?	“Relação íntima é termo dúbio; jovens agredidas nem sempre tem atividade sexual, mas isso não as impediria de responderem as questões sobre violência. Inclusive, o confronto das respostas poderia ser mais uma forma de aferição qualitativa, se algumas jovens agredidas não chegaram a ter relações sexuais com o agressor.” (E4)	66,5%	“[Relação íntima, para mim é] sexual. A impressão que dá, é porque assim, hoje a gente vê é que as pessoas têm muita preocupação com a palavra sexo. (…) Então usa-se, íntima, às vezes, até pra…deixar menos invasivo.” (P4)	1. Você já teve um relacionamento afetivo ou conjugal?
*(Since you were 16 years of age)*	(Desde os seus 16 anos de idade)	(Desde os seus 16 anos de idade)	ND	62,5%	ND	(Desde os seus 16 anos de idade)
*( ) Yes 1 ( ) No 0 ( Go to next section)*	( ) Sim 1 ( ) Não 0 ( Vá para a próxima seção)	( ) Sim 1 ( ) Não 0 ( Vá para a próxima seção)	ND	100%	ND	( ) Sim 1 ( ) Não 0 (Vá para a próxima pergunta)
*2. Have you been in an adult intimate relationship in the last twelve months?*	2. Você teve alguma relação íntima adulta nos últimos doze meses?	2. Você teve alguma relação íntima adulta nos últimos doze meses?	“Mesma questão sobre o uso de relações íntimas” (E2). “Sempre é o problema com relação íntima adulta” (E3). “A pergunta é excelente, importante, mas o termo ‘relações íntimas’ complica a resposta…” (E4)	75%	“Uma relação íntima você pode ter com uma pessoa que não seja de parceiro, pode ser uma pessoa que você conheceu e foi só aquele momento íntimo que você teve com ela, não necessariamente, você tem para frente algum outro contato. Mais envolvimento sexual mesmo.” (P8). “Se eu fiz sexo nos últimos 12 meses?” (P3)	2. Você teve algum relacionamento afetivo ou conjugal nos últimos doze meses?
*(Since you were 16 years of age)*	(Desde os seus 16 anos de idade)	(Desde os seus 16 anos de idade)	ND	100%	ND	(Desde os seus 16 anos de idade)
*( ) Yes 1 ( ) No 0 ( Go to question 6)*	( ) Sim 1 ( ) Não 0 ( Vá para a pergunta 6)	( ) Sim 1 ( ) Não 0 ( Vá para a pergunta 6)	ND	87,5%	ND	( ) Sim 1 ( ) Não 0 (Vá para a próxima pergunta)
*3. Are you currently in an intimate relationship?*	3. Você está em uma relação íntima adulta no momento?	3. Você está em uma relação íntima adulta no momento?	“A pergunta corresponde ao inglês, mas relação íntima é estranho; não seria melhor apenas perguntar: você está num relacionamento agora? Que no Brasil todo mundo entende?” (E4)	79%	“Pra mim é uma relação sexual (…) Então, tu entende que essa pergunta leva mais para uma questão sexual?” (P15)	3. Você está em um relacionamento afetivo ou conjugal no momento?
*( ) Yes 1 ( ) No 0 ( Go to question 5)*	( ) Sim 1 ( ) Não 0 ( Vá para a pergunta 5)	( ) Sim 1 ( ) Não 0 ( Vá para a pergunta 5)	ND	100%	ND	( ) Sim 1 ( ) Não 0 (Se não, por favor, vá para a pergunta 5)
*4. Are you currently afraid of your partner?*	4. Atualmente, você tem medo do seu parceiro ou parceira?	4. Atualmente, você tem medo do seu parceiro ou parceira?	ND	95,75%	ND	4. Atualmente, você tem medo do seu parceiro ou parceira?
*( ) Yes 1 ( ) No 0*	( ) Sim 1 ( ) Não 0	( ) Sim 1 ( ) Não 0	ND	100%	ND	( ) Sim 1 ( ) Não 0
*5. Have you been afraid of any partner in the last 12 months?*	5. Você teve medo do seu parceiro ou parceira nos últimos 12 meses?	5. Você teve medo do seu parceiro ou parceira nos últimos 12 meses?	ND	79%	ND	5. Você teve medo do seu parceiro ou parceira nos últimos 12 meses?
*( ) Yes 1 ( ) No 0*	( ) Sim 1 ( ) Não 0	( ) Sim 1 ( ) Não 0	ND	50%	ND	( ) Sim 1 ( ) Não 0
*6. Have you ever been afraid of any partner?*	6. Você já teve medo de algum parceiro ou parceira?	6. Você já teve medo de algum parceiro ou parceira?	ND	79%	ND	6. Você já teve medo de algum parceiro ou parceira?
*( ) Yes 1 ( ) No 0*	( ) Sim 1 ( ) Não 0	( ) Sim 1 ( ) Não 0	ND	100%	ND	( ) Sim 1 ( ) Não 0
*7. We would like to know if you experienced any of the actions listed below and how often it happened during the past twelve months. If you were not with a partner in the past twelve months, could you please answer for the last partner that you had. Please tick the appropriate box, which matches the frequency, over a twelve month period, that it happened to you.*	7. Gostaríamos de saber se você vivenciou alguma das ações listadas abaixo e com que frequência elas ocorreram nos últimos doze meses. Se você não teve um parceiro ou parceira nos últimos doze meses, por favor, responda à pergunta considerando o seu último parceiro ou parceira. Marque com um X a opção correta que corresponde à frequência com que a ação ocorreu com você nos últimos doze meses.	7. Gostaríamos de saber se você vivenciou alguma das ações listadas abaixo e com que frequência elas ocorreram nos últimos doze meses. Se você não teve um parceiro ou parceira nos últimos doze meses, por favor, responda à pergunta considerando o seu último parceiro ou parceira. Marque com um X a opção correta que corresponde à frequência com que a ação ocorreu com você nos últimos doze meses.	ND	87,5%	ND	7. Gostaríamos de saber se você vivenciou alguma das ações listadas abaixo e com que frequência elas ocorreram nos últimos doze meses. Se você não teve um parceiro ou parceira nos últimos doze meses, por favor, responda às perguntas considerando o seu último parceiro ou parceira. Marque com um X a opção correta que corresponde à frequência com que a ação ocorreu com você nos últimos doze meses.
*(Please tick one box on each line)*	(Por favor, marque uma das opções em cada linha)	(Por favor, marque uma das opções em cada linha)	ND	83,25%	ND	(Por favor, marque SOMENTE UMA das opções em cada linha)
*Actions*	Ações	Ações	ND	87,5%	ND	Ações
*How often it happened*	Com que frequência ocorreu	Com que frequência ocorreu	ND	100%	ND	Com que frequência ocorreu
*My Partner:*	Meu parceiro ou parceira:	Meu parceiro ou parceira:	ND	100%	ND	Meu parceiro ou parceira:
*Never*	Nunca	Nunca	ND	100%	“Eu entendi, zero que nunca fez e até o que é bem frequente.” (P4)	Nunca
*Only Onc*e	Somente uma vez	Uma vez^a^	ND	100%	ND	Uma vez
*Several Times*	Muitas vezes	Algumas vezes^a^	ND	100%	ND	Algumas vezes
*Once/Month*	Uma vez por mês	Uma vez por mês	ND	100%	ND	Uma vez por mês
*Once/Week*	Uma vez por semana	Uma vez por semana	ND	100%	ND	Uma vez por semana
*Daily*	Diariamente	Diariamente/Quase diariamente^a^	ND	100%	ND	Diariamente/Quase diariamente
*Told me that I wasn't good enough*	Falou que eu não era boa o suficiente	Falou que eu não era boa o suficiente	ND	100%	ND	Falou que eu não era boa o suficiente
*Kept me from medical care*	Impediu-me de obter tratamento médico	Impediu-me de obter tratamento médico	“Modificar a linguagem para coloquial: por exemplo, me impediu.” (E1)	100%	ND	Me Impediu de obter tratamento médico
*Followed me*	Seguiu-me	Seguiu-me	ND	87,5%	“Eu acho que me perseguiu é melhor [do que me seguiu]. A partir do momento que digo: está me perseguindo, aí ele tá me incomodando, o seguiu-me, não me incomoda, a palavra, né, não me incomoda. Não tem impacto.” (P8)	Me perseguiu
*Tried to turn my family, friends or children against me*	Tentou colocar minha família, amigos(as) ou filhos(as) contra mim	Tentou colocar minha família, amigos(as) ou filhos(as) contra mim	ND	100%	ND	Tentou colocar minha família, amigos(as) ou filhos(as) contra mim
*Locked me in the bedroom*	Trancou-me no quarto	Trancou-me no quarto	ND	95,75%	ND	Me trancou no quarto
*Slapped me*	Deu-me um tapa	Deu-me um tapa	ND	100%	“Como eu falei pra você, pela escrita, pela parte ortográfica está correta, mas a parte de compreensão o ‘me deu um tapa’ é mais forte do que dizer ‘deu-me um tapa’.” (P8)	Me deu um tapa
*Raped me*	Estuprou-me	Obrigou-me a ter relações sexuais contra a minha vontade	ND	100%	“Eu acho que é me obrigou. Sabe porque? Ela [violência] não tem classe social. Ela pega todas as camadas, desdes as pessoas mais simples a mais top, assim mais culturalmente privilegiada, financeiramente privilegiada, de raça privilegiada, não tem cor, não tem raça, não tem classe social.” (P14)	Me obrigou a ter relações sexuais contra a minha vontade
*Told me that I was ugly*	Falou que eu era feia	Falou que eu era feia	ND	100%	ND	Falou que eu era feia
*Tried to keep me from seeing or talking to my family*	Tentou me impedir de ver ou falar com a minha família	Tentou me impedir de ver ou falar com a minha família	ND	100%	ND	Tentou me impedir de ver ou falar com a minha família
*Threw me*	Empurrou-me	Jogou-me e derrubou-me	“O português corresponde, evidentemente, ao inglês; mas se o instrumento pretende ser abrangente, seria melhor o português coloquial; Exemplo: me impediu de ter tratamento médico; me trancou no quarto ou em casa; me obrigou a transar; me jogou e me derrubou no chão. Pode parecer trivial, mas a população não entende o português formal.” (E4)	91,5%	“Jogou-me e derrubou-me… tá bem estranho… Se você estiver falando de uma agressão é me jogou e me derrubou.” (P15)	Me jogou e me derrubou
*Hung around outside my house*	Ficou me vigiando do lado de fora da minha casa	Ficou me vigiando do lado de fora da minha casa	ND	95,75%	ND	Ficou me vigiando do lado de fora da minha casa
*Blamed me for causing their violent behaviour*	Culpou-me por ter causado seu comportamento violento	Culpou-me por ter causado seu comportamento violento	ND	100%	ND	Me culpou por ter causado seu comportamento violento
*Harassed me over the telephone*	Assediou-me pelo telefone	Assediou-me pelo telefone, internet ou redes sociais^a^	ND	95,75%	ND	Me assediou pelo telefone, internet ou redes sociais
*Shook me*	Chacoalhou-me	Chacoalhou-me	ND	95,75%	“Olha tem uma palavra bem estranha: chacoalhou, chacoalhou o quê? Pegou e me chacoalhou…me sacudiu eu acho que fica melhor.” (P9)	Me sacudiu
*Tried to rape me*	Tentou me estuprar	Tentou me forçar a ter relações sexuais contra a minha vontade	ND	100%	ND	Tentou me forçar a ter relações sexuais contra a minha vontade
*Harassed me at work*	Assediou-me no trabalho	Assediou-me no trabalho	ND	100%	ND	Me assediou no trabalho
*Pushed, grabbed or shoved me*	Empurrou-me ou agarrou-me	Empurrou-me ou agarrou-me	ND	100%	ND	Me empurrou ou me agarrou
*Used a knife or gun or other weapon*	Usou uma faca, um revólver ou outra arma	Usou uma faca, um revólver ou outra arma contra mim	ND	100%	ND	Usou uma faca, um revólver ou outra arma contra mim
*Became upset if dinner/housework was not done when they thought it should be.*	Ficou bravo/brava se o jantar ou afazer doméstico não foi feito do modo que ele(a) achava que deveria	Ficou bravo/brava se o jantar ou afazer doméstico não foi feito do modo que ele(a) achava que deveria	ND	91,5%	ND	Ficou bravo/brava se o jantar ou afazer doméstico não foi feito do modo que ele/ela achava que deveria
*Told me that I was crazy*	Disse-me que era louca	Disse-me que era louca	ND	100%	ND	Me disse que era louca
*Told me that no one would ever want me*	Disse-me que ninguém nunca vai me querer	Disse-me que ninguém nunca vai me querer	ND	100%	ND	Me disse que ninguém nunca vai me querer
*Took my wallet and left me stranded*	Pegou a minha carteira e deixou-me sem dinheiro	Pegou a minha carteira e deixou-me sem dinheiro	ND	95,75%	ND	Pegou a minha carteira e me deixou sem dinheiro
*Hit or tried to hit me with something*	Bateu ou tentou me bater com alguma coisa	Bateu ou tentou me bater com alguma coisa	ND	95,75%	ND	Bateu ou tentou me bater com alguma coisa
*Did not want me to socialise with my female friends*	Não quis que eu me encontrasse com minhas amigas	Não quis que eu me encontrasse com minhas amigas	“Na versão em português brasileiro pós-revisão da autora é equivalente à versão original em língua inglesa. Porém, não é equivalente à versão em português brasileiro, pois não mantém o sentido da expressão. Neste caso específico, acredito que do ponto de vista das experiências das mulheres, o acréscimo da palavra “amigos” na versão em português brasileiro contribui para a evidência (ou não) da ocorrência do fenômeno da VPI (o que atende ao item de pertinência “experimental”. (E5)	95,75%	ND	Não quis que eu me encontrasse com minhas amigas/amigos
*Put foreign objects in my vagina*	Colocou objetos estranhos na minha vagina	Colocou objetos estranhos na minha vagina contra a minha vontade	ND	100%	ND	Colocou objetos estranhos na minha vagina contra a minha vontade
*Refused to let me work outside the home*	Não deixou que eu trabalhasse fora de casa	Não deixou que eu trabalhasse fora de casa	ND	100%	ND	Não deixou que eu trabalhasse fora de casa
*Kicked me, bit me or hit me with a fist*	Chutou-me, mordeu-me ou me deu socos	Chutou-me, mordeu-me ou me deu socos	ND	100%	ND	Me chutou, me mordeu ou me deu socos
*Tried to convince my friends, family or children that I was crazy*	Tentou convencer meus amigos, família ou filhos(as) que eu era louca	Tentou convencer meus amigos, família ou filhos(as) que eu era louca	ND	100%	ND	Tentou convencer meus amigos(as), família ou filhos(as) que eu era louca
*Told me that I was stupid*	Disse-me que era burra	Disse-me que era burra	ND	100%	ND	Me disse que eu era burra
*Beat me up*	Bateu em mim	Bateu em mim	ND	100%	ND	Bateu em mim

ND: nada a declarar; VPI: violência por parceiro íntimo; E1,E2…: especialista 1, especialista 2…; P3,P4…: entrevistada 3, entrevistada 4…

a As alternativas foram sugeridas pela desenvolvedora do instrumento a partir da revisão da *Composite Abuse Scale*, feita em 2016 (Ford-Gilboe et al.^26^, 2016).

Na etapa (b), algumas divergências entre os dois tradutores (T1 e T2), foram discutidas entre a equipe de pesquisa (mestranda e orientadores) em reuniões para comparação, análise e síntese entre as duas versões traduzidas e o instrumento original. Ainda houve contribuição dos tradutores para dirimir dúvidas, obtendo-se assim a VR1 (c), apresentada no Quadro 1.

Na etapa (d) a VR1 foi retrotraduzida de maneira cega, sem que o retrotradutor conhecesse o instrumento original, nem tampouco se comunicasse com os tradutores. O retrotradutor enviou alguns questionamentos para a equipe coordenadora da pesquisa sobre palavras que não tinham tradução literal em português, mas que tinham uma conotação de intensidade diferente em língua inglesa (ex. item 17 – “*Pushed, grabbed or shoved me”*) ou neutralidade de gênero no instrumento em inglês (p. ex. “*partner*”), que implica em flexão de gênero em português (“parceiro”/”parceira”).

A versão retrotraduzida foi enviada à desenvolvedora para análise (etapa e). Dúvidas que surgiram nas etapas anteriores também foram apontadas à desenvolvedora, em reunião para buscar consensos. A opinião da desenvolvedora foi fundamental para manter a fidedignidade ao instrumento original, mas ao mesmo tempo mantendo-se linguagem atualizada e contextualizada ao cenário brasileiro. Um dos ajustes nessa etapa incluiu a adaptação do item 7, “*Raped me*” por “Obrigou-me a ter relações sexuais contra a minha vontade”, visto que muitas mulheres casadas (ou em um relacionamento) não percebem relações sexuais forçadas dentro do casamento/relacionamento como estupro. Outra adaptação foi no item 25 (“*Put foreign objects in my vagina*”) que ficou “Colocou objetos estranhos na minha vagina contra a minha vontade”, para diferenciar de relações consensuais. A desenvolvedora também sugeriu atualização de alguns itens a partir de uma revisão recente da CAS^26^, como no item 13, “*Harassed me over the telephone”*, que passou a incluir também Internet e redes sociais. Ao final dessa etapa (e), chegou-se à VR2, detalhada no Quadro 1.

Na etapa (f) participaram seis especialistas (E), docentes em universidades de diferentes regiões do país, cujos perfis constam no Quadro 2. As áreas de formação foram bastante diversas, incluindo Saúde, Psicologia, Ciências Humanas e Exatas, possibilitando olhares multidisciplinares sobre a tradução e adaptação transcultural da CAS.

**Quadro 2:** Perfil de especialistas (E) participantes da etapa de análise por especialistas (etapa f).

Especialista	Gênero	Graduação	Titulação máxima
E1	Mulher	Enfermagem e Filosofia	Pós-doutora em Tecnologia
E2	Mulher	Ciências com Habilitação em Matemática	Pós-doutora em Estudos Interdisciplinares sobre Mulheres, Gênero e Feminismos
E3	Homem	Psicologia	Doutor em Saúde Coletiva
E4	Mulher	Medicina	Doutora em Medicina Preventiva
E5	Mulher	Ciências Sociais e Políticas	Doutora Interdisciplinar em Ciências Humanas
E6	Mulher	Psicologia	Doutora em Saúde Coletiva

E1,E2…: especialista 1, especialista 2…

Quatro de seis especialistas problematizaram o termo “relações íntimas adultas” e o motivo de não termos utilizado “relações sexuais” ou “relações afetivo-sexuais”. Sobre o termo “relações íntimas adultas”, o próprio instrumento explica seu significado, conforme pode ser observado no Quadro 1 (VR2): “*Por relações íntimas adultas, nos referimos a esposo/esposa, parceiro/parceira ou namorado/namorada por um período maior que um mês.*” O comitê chamou atenção para que no Brasil, diferentemente de outros países, mulheres de 16 anos não são consideradas adultas, visto que o instrumento pode ser preenchido por pessoas a partir dos 16 anos. Desse modo, retiramos a palavra “adultas”, mas mantivemos o termo “relações íntimas*”* por conta da explicação desse termo, que vem logo a seguir no próprio instrumento.

Especialistas também demonstraram preocupação com a compreensão dos itens e comandos para preenchimento adequado, reforçando que a linguagem deveria ser mais coloquial que acadêmica. Sinalizaram a necessidade da troca de ênclises por próclises (Ex: “*derrubou-me*” por “*me derrubou*”); e necessidade de flexão de gênero, como no item 24 – “*Did not want me to socialise with my female friends*” por “*Não quis que eu me encontrasse com minhas amigas/amigos*”, uma vez que é comum na sociedade brasileira mulheres também terem amigos homens.

A partir das considerações de especialistas e reuniões de conciliação entre a equipe, chegou-se à VR3, submetida então às entrevistas cognitivas com mulheres da CMB, com o intuito de avaliar se os itens foram compreendidos como esperado. Das 15 participantes dessa etapa, sete se identificaram como brancas, quatro como negras e quatro não souberam responder; 11 se identificaram como heterossexuais, duas como bissexuais e duas como lésbicas, todas elas mulheres cis. Quanto à renda, foram três participantes com renda familiar de até um salário mínimo (SM), duas entre um e dois SM, uma entre dois e três SM, e nove de três ou mais SM. Oito participantes eram as principais responsáveis pela renda familiar. Quanto à escolaridade, duas possuíam ensino médio, quatro ensino superior e nove com pós-graduação.

Após disponibilizar uma via impressa (VR3), perguntou-se o que entendiam por cada item. Algumas dúvidas emergiram, representadas no Quadro 1 (coluna “Entrevistas cognitivas”). O termo mais problemático foi “relação íntima”, que já havia sido problematizado na etapa de especialistas. A compreensão das mulheres sobre relação íntima, geralmente discordava do conceito original, afirmando que “relação íntima” era entendido como “relação sexual”. A entrevistadora percebeu que mesmo após a leitura do trecho explicativo da CAS que descreve o que é considerado como uma relação íntima, as participantes permaneceram associando a expressão ao ato sexual. Até mesmo as participantes que, após a leitura do conceito, entenderam que se refere a uma relação de namoro, casamento ou de parceria íntima, ao longo da entrevista associavam novamente a relação íntima ao ato sexual. Essa interpretação ocorreu em todas as perguntas que continham esse termo. A participante P3, quando solicitada que respondesse qual era a sua compreensão sobre a pergunta “Você teve alguma relação íntima nos últimos doze meses?”, respondeu imediatamente: “*Quer saber se eu fiz sexo nos últimos 12 meses?*”.

As críticas das participantes convergiam com os apontamentos dos especialistas e, considerando as sugestões deles, a entrevistadora indagou se a pergunta fosse reformulada para “*Você teve algum relacionamento afetivo ou conjugal…?”* As participantes então interpretaram que, portanto, sim, a pergunta estaria relacionada a namoro, casamento ou parceria afetiva. Também concordaram unanimemente que a nova formulação melhorava o entendimento, como exemplifica P4: “*Nessa pergunta é num tom, assim, diferente, sabe, eu não consigo ver o lado sexual como nas outras. Eu consigo ver como algo tipo, há um namoro.”*

Outro questionamento levantado pelas participantes foi sobre a linguagem do instrumento e sua equivalência experiencial. A maioria recomendou uma linguagem mais coloquial, sem o recurso da ênclise, como já apontado pelos especialistas. P4 exemplifica: “*Me deu um tapa, é linguagem popular… é a linguagem que mais as pessoas usam…”* Quando perguntado à P15 se seria mais inteligível “Me jogou e me derrubou” ao invés de “Jogou-me e derrubou-me”, a participante respondeu: *“Se você estiver falando de uma agressão é… me jogou e me derrubou.”* Com base nisso, foram substituídas todas as ênclises por próclises, conforme consta na versão final do instrumento, cientes de que não correspondem à ortografia correta da língua portuguesa, mas atentando-se às necessidades de compreensão e interpretação da população geral.

Algumas participantes também questionaram o item 3 (“Seguiu-me”), pois consideraram que não passava a ideia de violência ou poderia gerar duplo sentido, pensando no contexto das redes sociais (“me seguiu no facebook”). Algumas sugeriram que “Me perseguiu” seria mais adequado. Mesmo as que não sugeriram a modificação do termo, ao explicar o que compreendiam sobre ele utilizavam a palavra “perseguir”: “*O parceiro me perseguiu”* (P10); *“Alguém está me perseguindo”* (P11). O item foi então alterado para “Me perseguiu”.

No item 14 (“Chacoalhou-me”), 11 das 15 participantes consideraram que seria melhor substituir por “Me sacudiu”. Ainda que elas compreendessem o significado do termo “Chacoalhou-me”, a palavra “Sacudir” soaria melhor, como exemplifica (P6): “*Eu acho que me sacudiu fica melhor…”.* P14 adiciona que: “*me sacudiu, acho que qualquer pessoa consegue pegar*”. Ela considera, portanto, mais acessível à compreensão de pessoas de diferentes escolaridades.

Nos demais itens não houve apontamentos discordantes, havendo compreensão consensual. Algumas participantes destacaram a relevância do instrumento para mulheres brasileiras, verbalizando que a CAS está compatível com o cenário brasileiro, é prática, útil e pode ser inclusive necessária. Uma das profissionais comenta nesse sentido:

“Q*uando elas chegam aqui* [CMB]*, isso aqui tudo, bem dizer, já ocorreu. Muitas delas, já ocorreram. E ela, realmente, ela não se vê. E a partir do momento em que ela responder isso aqui, ela vai dizer assim: Meu Deus! É isso o que acontece comigo*”. (P8)

Ela, então corrobora a compatibilidade da CAS com a realidade das mulheres que procuram a CMB e que o instrumento pode, inclusive, ajuda-las a visibilizar e compreender as situações de violências a que estão submetidas em suas relações.

Alguns itens chamaram atenção dos pesquisadores, como “2 – Impediu-me de obter tratamento médico”, segundo revelou P5, “*Isso é muito frequente, os parceiros não permitem que elas frequentem ou façam acompanhamento*”. Isso sinaliza desafios para o campo da saúde no cuidado dessas mulheres diante de comentários de profissionais que trabalham diariamente com mulheres em situação de violências. Assim, a equipe de pesquisa considera um achado relevante para a compatibilidade do instrumento com o contexto cultural brasileiro, ainda que tenha sido criado em outro país.

Quanto ao design do instrumento e preenchimento dos itens, algumas participantes elogiaram pela objetividade e facilidade de compreensão, como P6: *“Eu acho que está ótimo assim. Bem melhor do que aquelas avaliações, escalas, que eu acho terrível, do escolhe parcialmente, concorda parcialmente. Aqui está bem claro e objetivo”.* A participante se refere a alguns instrumentos que utilizam escalas do tipo Likert^45^ como método para o preenchimento. Por outro lado, duas participantes fizeram críticas ao design do instrumento, cujo destaque foi importante para incorporar na versão final. Trata-se da necessidade do maior espaçamento entre os itens para facilitar a visualização, assim como o uso de linhas ou diferentes tons de sombreamento para separar uma pergunta da outra.

## DISCUSSÃO

A implementação do protocolo^32^ de tradução e adaptação transcultural da CAS resultou na “CAS Versão Português Brasileiro”, adaptada ao contexto das mulheres brasileiras e com equivalência idiomática, semântica, conceitual e experiencial^33,34^. O instrumento pode ser autoaplicável, gerando um escore que permite classificar a mulher como vivendo em situação de violência por parceiro íntimo (escore ≥ 7 pontos), numa faixa de escore que varia de 0 a 150 pontos^18,19^. A pesquisa de campo destacou que a escala pode ajudar as mulheres a visualizarem situações de abuso vivenciadas em seus relacionamentos, auxiliando-as a perceberem a VPI.

As principais adaptações realizadas ao longo do processo consistiram na modificação de alguns termos, como substituição de “relações íntimas” por “relações afetivas ou conjugais” pois têm equivalência melhor ao construto original, que se refere a relacionamentos afetivos com cônjuges com no mínimo um mês de duração, e não somente relações sexuais. Uma significativa adaptação foi a troca de ênclises por próclises em 20 itens, cientes de que tal mudança não contempla a ortografia segundo as normas da língua portuguesa, mas atende a uma demanda das potenciais usuárias, facilitando sua compreensão. Outra adaptação significativa foi a incorporação de linguagem neutra, como por exemplo parceiro/parceira, permitindo que o instrumento seja usado por mulheres em relacionamentos hetero, bi ou homoafetivos.

Outro aspecto foi a substituição do termo “*Rape me*”, do original em inglês, por “Me obrigou a ter relações sexuais contra a minha vontade”, levando em consideração achados na literatura demonstrando que mulheres não acreditam que possam ser estupradas dentro de relacionamentos afetivos/conjugais e, portanto, não associam esse tipo de relação sexual à violência por parceiro íntimo^46–48^. Da mesma forma, adaptou-se também o item 15 “*Tried to rape me*” para “Tentou me forçar a ter relações sexuais contra a minha vontade”.

Questões adicionais problematizadas pelo comitê de especialistas se relacionaram aos tipos de relacionamentos, sugerindo incluir outros vínculos e práticas íntimas (como poliamor, por exemplo), assim como relações de amizade. Todavia, preocupados em manter a fidedignidade ao instrumento original, a equipe de pesquisa reflete que a CAS apresenta essa limitação por não incluir esses outros tipos de vínculos. No entanto, essa é uma lacuna que não será possível resolver neste estudo, sendo necessário um outro tipo de pesquisa ou criação de novo instrumento que aborde especificamente essas questões. Mesmo considerando importante a reflexão sobre os diversos tipos de vínculos existentes em relações íntimas, um dos propósitos da tradução e adaptação transcultural de uma escala para outro idioma/cultura é a possibilidade de comparar os resultados obtidos a partir de sua aplicação em outros países/idiomas/culturas. Portanto, o instrumento não pode sofrer alterações substanciais, que não permitam a comparabilidade das respostas entre os idiomas. No caso da CAS, as instruções são explícitas no que se refere ao preenchimento do instrumento, que deve ser feito pensando na pessoa que perpetrou a violência e que está há pelo menos um mês em uma relação (afetivo ou conjugal) com quem está preenchendo a escala.

A etapa de entrevistas cognitivas foi fundamental no processo de tradução e adaptação transcultural para investigar a compreensão das mulheres que serão as potenciais usuárias da CAS. Collins^49^ explica que as perguntas dessa etapa, particularmente quando aplicadas no contexto do instrumento, permitem determinar se os entrevistados podem compreender o conceito ou tarefa da pergunta e se fazem isso de maneira consistente e da maneira pretendida pelo pesquisador. Assim, a condução dessa etapa na CMB, que atende exclusivamente mulheres em situação de violência, demonstrou ser uma estratégia efetiva para adaptação da CAS às necessidades das mulheres brasileiras. Tanto conteúdo quanto significados e formato de apresentação do instrumento foram avaliados por potenciais usuárias ou por profissionais que as atendem diariamente.

Em relação ao layout, ainda que somente duas dentre as 15 participantes dessa etapa tenham criticado o design, percebe-se em suas falas a importância de absorver as críticas a fim de tornar o preenchimento do instrumento o mais intuitivo e fácil possível, já que a CAS foi idealizada para ser autopreenchida pelas mulheres.

O estudo teve como principais limitações o fato de as entrevistas cognitivas terem sido conduzidas em apenas um único local, na Região Sul do país e sem participação de mulheres com baixa escolaridade. É possível que mulheres com menor grau de instrução tenham alguma dificuldade no preenchimento e que nuances idiomáticas regionais de outras áreas do país possam não ter sido suficientemente incorporadas ao instrumento. Portanto, recomenda-se em estudos futuros que a CAS brasileira seja aplicada a um número maior de mulheres, com escolaridade mais diversificada. Por outro lado, a etapa com especialistas incluiu pesquisadoras de diferentes regiões do país, que foram convidadas a exercer sua análise pensando no público diverso de mulheres brasileiras (regionalismos, graus de escolaridade, faixa etária etc.). E de fato foram feitos apontamentos nessa direção, como a simplificação da linguagem para facilitar a compreensão das respondentes.

Em síntese, as participantes deram veredito de pertinência para a CAS Versão Português Brasileiro. Além da avaliação positiva quanto à compreensão linguística, para elas o instrumento incorporou aspectos sobre a violência por parceiro íntimo que também dizem respeito à realidade brasileira e, portanto, precisam ser monitorados. Assim, o processo de tradução e adaptação transcultural foi concluído com êxito, sendo disponibilizada a CAS Versão Português Brasileiro para utilização da comunidade acadêmica brasileira. Cabe apontar a necessidade de o instrumento ter suas propriedades psicométricas avaliadas em estudos futuros. Desse modo, poderá ser disponibilizada de acordo com os mais altos padrões metodológicos e de rigor científico para ampla utilização no Brasil.
